# Deep Vein Thrombosis and Pulmonary Embolism in the Setting of Mycoplasma Infection

**DOI:** 10.1155/2020/8708417

**Published:** 2020-09-06

**Authors:** Sandal Saleem, Brian Berman

**Affiliations:** ^1^Department of Pediatric Emergency Medicine, Beaumont Health Royal Oak, 3601 W. 13 Mile Rd., Royal Oak, MI 48073, USA; ^2^Oakland University William Beaumont School of Medicine, 586 Pioneer Dr Ste 428, Rochester, MI 48309, USA

## Abstract

Thirteen-year-old female twins presented one week apart with documented *Mycoplasma pneumoniae* respiratory infection. Each developed venous thrombosis and pulmonary emboli in association with transient self-limited para-infectious anti-phospholipid antibodies. Comprehensive evaluation revealed no identifiable genetic prothrombotic variables. Both children recovered after receiving antibiotics and anticoagulation therapy. Thrombotic complications associated with *Mycoplasma pneumoniae* infections are rare, particularly in children; the occurrence of this complication in identical twins has not been previously reported.

## 1. Introduction

*Mycoplasma pneumoniae* is a common pathogen causing community-acquired pneumonia in children [1, 2]. Extra pulmonary manifestations such as hematologic, neurologic, and mucocutaneous complications associated with *Mycoplasma* are well recognized. Thromboembolic events in association with mycoplasma have been reported. An analysis published in 2016 cited 9 pediatric cases of *Mycoplasma*-associated thromboembolic event [3]. All of these patients had anti-phospholipid antibodies [4]. We report identical twins with *Mycoplasma pneumoniae* infection complicated by pulmonary emboli in association with anti-phospholipid antibodies.

## 2. Case Reports

### 2.1. Patient 1

A 13-year-old Caucasian female presented with cough and fever of 1-week duration. Chest X-ray revealed left lower lobe segmental pneumonia. The patient was admitted to the hospital after she failed to improve. Intravenous ceftriaxone was added to azithromycin. Blood culture was negative, and sputum culture grew normal flora. Initial laboratory tests showed the following: hemoglobin of 12.2 (12–15 g/dl), white blood cell count of 4.5 (4.3–13.5 thous/*μ*l), and platelet count of 236 (150–500 thous/*μ*l). *Mycoplasma pneumoniae* PCR was positive followed by elevated specific Ig M being 4.10 (<0.9) and IgG being 0.88 (<0.9). On day 3 of hospital admission, she developed increasing respiratory distress. Antibiotics coverage was broadened. Chest CT with contrast showed multifocal pneumonia with bilateral parapneumonic effusions. The patient ultimately required BiPAP for respiratory support and was transferred to the pediatric intensive care unit (PICU).

On day 2 of admission to the pediatric intensive care unit, she complained of left calf pain with mild swelling. US doppler revealed deep vein thrombosis (DVT) in left gastrocnemius, posterior tibial (left), and popliteal and peroneal veins, and chest CT was reread as showing filling defects involving segmental branches to the right middle lobe pulmonary artery and right lower lobe segmental pulmonary arteries. She was started on enoxaparin at a dose of subcutaneous 60 mg (1 mg/kg/dose) twice daily. Echocardiogram was significant for elevated right ventricle (RV) systolic pressure. Anti-phospholipid antibody studies demonstrated an elevated anticardiolipin IgG of 86 (0–14.9 GPL), IgM of 120.7 (0–12.4 MPL), normal IgA of <9.5 (0–11.9 APL), and anti-beta-2-glycoprotein IgG/IgM of <9.5/21.9 (<9.5/21.9 GGU/SMU). DRVVT was elevated to 54 with a normalized ratio of 1.3. On PICU day 4, antifactor Xa level was subtherapeutic and enoxaparin dose was adjusted to 70 mg after which the therapeutic level of antifactor Xa of 0.7 U/ml (0.6–1 U/ml) was achieved. On PICU day 5, based on her clinical improvement, antibiotics were weaned. She continued to improve and was discharged after 9 days.

At follow-up, her enoxaparin was switched to single daily dose (120 mg) to achieve a therapeutic level of 1.3 U/ml (1-2 U/ml) for a duration of 3 months. Symptoms completely resolved, and she returned to normal and full activities.

### 2.2. Patient 2

The 13-year-old Caucasian identical twin of the patient cited above presented to the emergency department with cough of 9-day duration and left leg pain of 2-day duration. She was seen a week prior by her primary care doctor who started azithromycin. Two days later, she was noted to be hypoxic (89% on room air), tachycardiac (131 bpm), and febrile (38.4°C) in the emergency department. Lung exam showed diminished breath sound on the left, and left calf tenderness was noted. Initial laboratory tests showed the following: hemoglobin of 11.9 g/dl (12–15 g/dl), white blood cell count of 9.6 thous/*μ*l (4.3–13.5 thous/*μ*l), platelet count of 259 thous/*μ*l (150–500 thous/*μ*l), lactate of 1.2 mmol/l (0.5–2.2 mmol/l), and BNP of <10 pg/ml (0–100 pg/ml). *Mycoplasma pneumoniae* PCR was negative followed by elevated specific IgM and IgG levels of 4.39 (<0.9) and 2.74 (<0.9), respectively. Echocardiography was unremarkable. Anti-nuclear antibodies were normal. Anti-phospholipid antibody studies demonstrated anticardiolipin IgG of 13.7 (0–14.9 GPL), IgM of 28.8 (0–12.4 MPL), normal IgA of <9.5 (0–11.9 APL), and anti-beta-2 glycoprotein IgG/IgM <9.5/<9.5 (<9.5/21.9 GGU/SMU). DRVVT was elevated to 54 sec (0–41 sec) with a normalized ratio of 1.3. A chest X-ray revealed right perihilar interstitial infiltrates. Antibiotic coverage was broadened. Doppler ultrasound for deep vein thrombosis of the lower extremity showed normal venous flow dynamics. CT angiography demonstrated multiple bilateral pulmonary emboli in segmental distribution of bilateral upper lobes, right middle lobe, and left lower lobe, along with dense consolidation of the right lower lobe. She was admitted to the PICU where she was started on an intravenous heparin drip.

On day 2 of hospital admission, she was switched to enoxaparin 60 mg twice daily (1 mg/kg/dose). Initial antifactor Xa level was subtherapeutic. Dose was adjusted to 80 mg twice daily, and a therapeutic level of antifactor Xa of 0.7 U/ml (0.6–1 U/ml) was achieved. She demonstrated gradual improvement and was discharged home on day 6.

At follow-up, she was switched to single daily dose of enoxaparin (120 mg) with a therapeutic antifactor Xa level of 1.5 U/ml (1-2 U/ml) for a duration of 3 months. She had complete resolution of symptoms and returned to normal and full activities.

## 3. Discussion

There are acquired and inherited factors leading to a hypercoagulable state [5]. Acquired factors that promote coagulation pathways include surgery, pregnancy, hormonal replacement therapy, estrogen containing oral contraceptive, malignancy, inflammation, infection, and heparin-induced thrombocytopenia. Genetic factors such as factor V Leiden and prothrombin G2021 A mutation are identified in a variable percentage of patients depending on demographics.

*Mycoplasma pneumoniae*-associated hypercoagulable state has been described to be secondary to endothelial damage leading to release of proinflammatory cytokines and activation of procoagulant pathways [2, 3]. Pulmonary emboli are the third leading cause of mortality associated with mycoplasma infection if not diagnosed and treated promptly [3].

Anti-phospholipid antibodies are found to be elevated in 1–5% of the healthy population [6]. They can also be elevated in bacterial infection, viral infections, and autoimmune diseases (i.e., lupus) [6, 7]. Elevated anti-phospholipid antibodies have also been reported after immunization [8, 9]. The mechanism by which thrombosis is precipitated in patients with anti-phospholipid antibodies remains unclear.

Most patients with infectious causes of elevated aPL antibody return to normal levels within 2 or 3 months [10]. Thromboembolic events in association with elevated aPL in the setting of infection are difficult to differentiate from other causes. For both patients, prothrombotic and genetic laboratory evaluations were initiated after enoxaparin was discontinued to identify potential genetic prothrombotic variables in these identical twins. Laboratory studies revealed resolution of elevated anti-phospholipid antibodies (anticardiolipin IgA, IgG, IgM, beta-2-glycoprotein 1 IgG and IgM, and DRVVT). The genetic evaluation was normal for factor V Leiden, prothrombin gene mutation, fibrinogen, thrombin time, factor VIII, protein C activity, protein S activity, plasminogen and plasminogen activator inhibitor activity, homocysteine level, ANA screen, dsDNA, and lipoprotein A. Although heterozygosity for MTHFR C677 T was found in both patients, it was not thought clinically relevant in the context of normal homocysteine level [11].

## 4. Conclusion

Our report documents the unique occurrence of transient para-infectious anti-phospholipid antibodies provoked by *Mycoplasma* infection leading to venous thrombosis and pulmonary embolism in identical female twins. We found no identifiable genetic prothrombotic variables. This does not rule out an underlying unknown genetic factor promoting thrombosis in these identical twins.

## Data Availability

No data were used to support the findings of this study.
